# Openable artificial intestinal tract device integrated with a permeable filter for evaluating drug permeation through cells

**DOI:** 10.1038/s41598-023-38522-x

**Published:** 2023-07-17

**Authors:** Satoshi Konishi, Shingo Ishibashi, Shiho Shimizu, Keita Watanabe, Rodi Abdalkader, Takuya Fujita

**Affiliations:** 1 Department of Mechanical Engineering, College of Science and Engineering, Ritsumeikan University, Kusatsu, 525-8577 Japan; 2 Graduate Course of Science and Engineering, Ritsumeikan University, Kusatsu, 525-8577 Japan; 3 Ritsumeikan Advanced Research Academy, Kyoto, 604-8520 Japan; 4 Ritsumeikan Global Innovation Research Organization, Ritsumeikan University, Kusatsu, 525-8577 Japan; 5 Graduate School of Pharmaceutical Sciences, Ritsumeikan University, 1-1-1 Noji-Higashi, Kusatsu, Shiga 525-8577 Japan; 6 Department of Pharmaceutical Sciences, Ritsumeikan University, 1-1-1 Noji-Higashi, Kusatsu, Shiga 525-8577 Japan

**Keywords:** Lab-on-a-chip, Biomedical engineering

## Abstract

Organs-on-chips using cultured cells have been developed and applied for evaluating in vitro biological phenomena. We previously reported an openable artificial intestinal tract system, as an in vitro model of the small intestine, for in vitro drug screening. The intestinal tract device could be transformed using an integrated artificial muscle actuator. An initial flat state was suitable for cell culture, and the transformed tubular structure was used as a fluidic channel for perfusion tests. The previously developed intestinal tract system could be used to evaluate drug absorption by cells through perfusion testing. This study presents an improved artificial intestinal tract system for analysis of drug permeation, in addition to absorption. Permeable filters were integrated into the intestinal tract device. Integration of additional filters into the design of the existing artificial muscle actuator was accomplished by considering device performance and available filter locations. Filter permeability was evaluated by perfusion testing. MDCK-II cells were cultured on the device and visually and electrically evaluated. The openable device, equipped with new functions for further pharmacokinetic analysis, could perform and evaluate drug disposition using cultured cells. We anticipate that the improved, openable organ-on-a-chip device system will contribute to advances in in vitro drug screening technology.

## Introduction

The organ-on-a-chip (OoC) platform, based on tissue engineering and lab-on-a-chip (LOC) technology, has shown remarkable progress^1,2^. In vitro drug screening is a promising application of OoC, as an alternative to whole-animal experiments. Various organs have been reproduced on a chip by culturing^3–8^. Neurons were cultured and analyzed on a microelectrode array chip for reducing animal experiments based on brain tissue samples^3^. Recently, microfluidic-based LOC technology enabled the integration of various functions for culturing, stimulating, and analyzing cells on a chip. Culture media and reagents can be supplied and exchanged via microchannels for cultured cells growing in a microchamber. Consequently, OoCs of various organs, such as the heart^4^, lungs^5^, gut^6^, liver^7^, and skin^8^, have been developed. Furthermore, studies on the mutual relationship between tissues and organs have commenced for developing more realistic in vitro models, based on individual OoCs.

Historically, conventional planar culture of cells and tissues on dishes and biochips was popular. Transwell^®^ cell culture insert (Corning Incorporated Life Sciences, Corning, NY, USA) is commonly used for estimating intestinal absorption and drug distribution^9,10^. However, conventional tools, such as dishes and Transwell^®^, cannot reproduce hydrodynamic conditions, including shear stress by flow.

Cell cultures at the bottom of a microchannel mimic the blood vessels and intestinal tract, and they have been used for perfusion tests^6,11–18^. For example, the gut-on-a-chip^6^ having upper and lower microchannels, separated by a central partitioning wall, was designed for more complicated analyses using substance permeation through the wall. Colon carcinoma cell line (Caco-2) cells were cultured on top of a central permeable membrane, which was used as a partitioning wall for substance exchange, through the boundary between two microchannels.

Studies continue to utilize cell cultures at the bottom of microchannels, where three-dimensional (3D) structures are provided. Planar cell culture conditions are fundamentally different from in vivo 3D tissue structures, and 3D cell cultures have been developed for OoCs for overcoming the drawbacks of planar cell culture^19–21^. For example, 3D intestinal organoids expressing villus-like structures and crypt-like proliferative zones were created by promoting intestinal growth, morphogenesis, and cytodifferentiation^19^. A technique for fabricating microscale 3D hydrogel scaffolds, using a microscale collagen structure, was demonstrated for mimicking human intestinal villi^20^. A small intestinal bioreactor, based on 3D printing and polymeric scaffolds, was reported for mimicking gut surface topography and fluid flow dynamics in the intestine^21^.

Most approaches to 3D structures for OoCs have dealt with cultured cell topography on a chip. We previously developed an openable artificial intestinal tract device, on which were cultured Caco-2 cells, to serve as an in vitro tubular tissue structure^22–24^. The openable artificial intestinal tract device could be transformed with an integrated artificial muscle actuator, and cells could be cultured on a flat surface, in the same manner as before, without changing conditions. The device could transform its shape into a tubular structure using integrated pneumatic balloon actuators (PBAs). The PBAs bend upwards by increasing internal pressure. The cultured cells on the surface of the device were deformed together with the device, and were thus distributed on the internal surface of the tubular structure. The tubular structure could be used for perfusion tests in the presence of liquid media and the drug. This openable artificial intestinal tract device, with a typical diameter of 1 mm, was used for an intestinal drug absorption assay^22^. Subsequently, the effect of an unstirred water layer, which creates a variation between in vitro and in vivo experiments, was evaluated using the openable artificial intestinal tract device^23^. Furthermore, the morphological transformation of a tubular structure as a scaffold substrate for cultured cells was demonstrated for coordinated motions of PBAs^24^. Peristaltic motions could be mimicked by the sequential independent motions of three sections of an array of PBAs.

**Figure 1:**
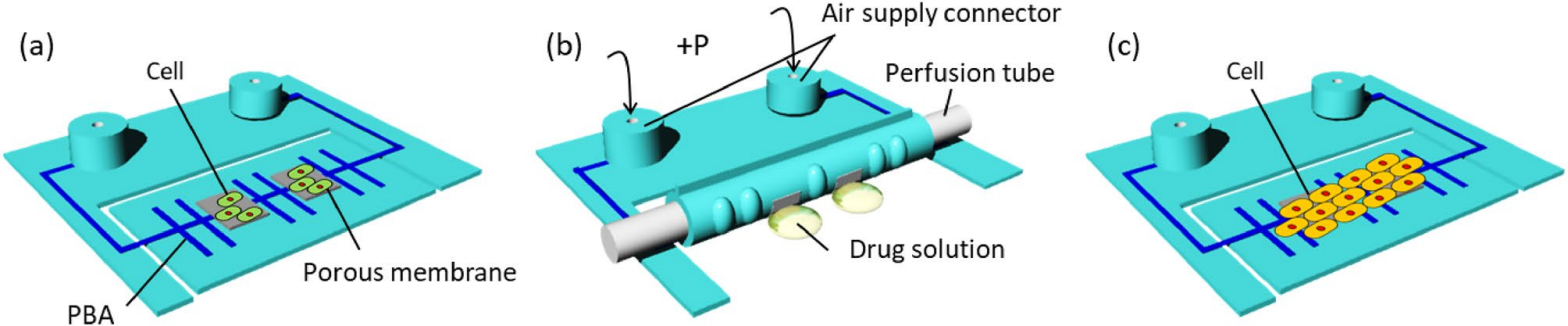
Schematic view of the artificial intestinal tract device for drug permeation test. (**a**) Cells are seeded on the device composed of PBAs and porous membrane. (**b**) After applying air pressure through air supply connectors, the tubular device is connected to the perfusion tube. The perfusion test under kinetic conditions is conducted by sampling the permeated drug through the porous membrane. (**c**) Cells are observed by transforming to a flat state.

The openable artificial intestinal tract device^22,23^ has been used for the evaluation of drug absorption. Pharmacokinetic analysis is used for evaluating absorption, distribution, metabolism, and excretion of drugs. The previous intestinal tract device could principally evaluate drug absorption by cells because the device was an impermeable polydimethylsiloxane (PDMS) structure. Transwell^®^ cell culture inserts have a permeable membrane for estimating intestinal absorption and drug distribution. In this study, we designed a permeable structure for the openable artificial intestinal tract device to enable further pharmacokinetic analysis, especially drug distribution through permeation (Fig. 1). Figure 1 shows the cell preparation and evaluation of intestinal absorption and drug distribution, using the developed system composed of the openable intestinal tract device with integrated filters. A permeable membrane is machined, assembled, and embedded into the device. We anticipate that the improved openable device as a platform for OoC, with additional functions of drug permeation, will be useful and efficient for cell-based screening owing to its applications for in-depth pharmacokinetic analysis.

## Materials and methods

### Fabrication of devices

The intestinal tract device, mounted on an acrylic system platform structure, was made largely of polydimethylsiloxane (PDMS) (Silpot 184, Dow Silicones Corp., Midland, Michigan, USA). The prepolymer ratio was adaptively controlled by considering the required elasticity of each structure. Prepolymer ratios of 8:1 and 12:1 were used for the inflatable layer and base layer, respectively. PDMS microstructures were cast using a mold that was patterned by photolithography of thick negative photoresist (Product number: SU-8 3050; Newton, MA, USA). Molded PDMS structures were bonded and integrated for completing the fabrication process.

**Figure 2:**
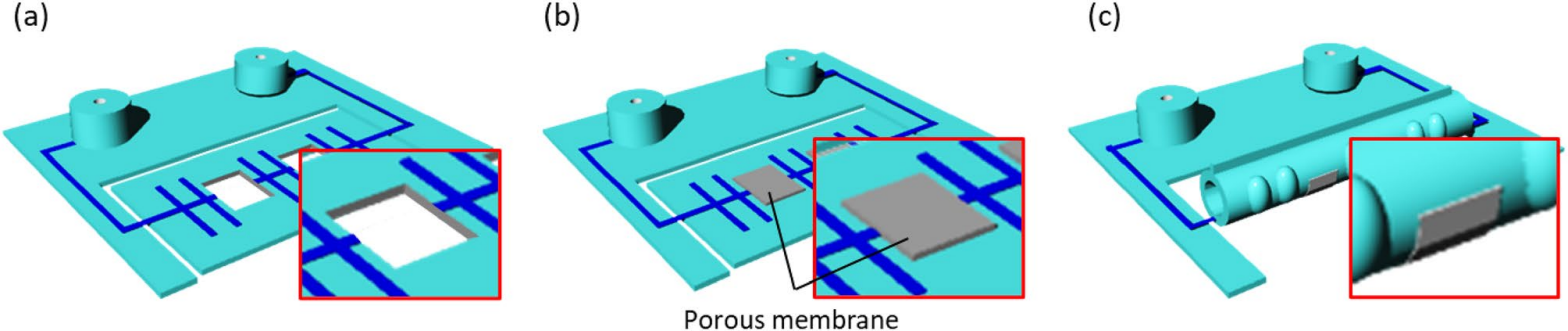
Fabrication process of the artificial intestinal tract device for drug permeation test. The basic fabrication process of the device resembles the conventional process for the device without filters. Filters are integrated into openings of 4 mm^2^ by gluing PDMS and heating.

Figure 2 shows the fabrication process of the intestinal tract device with integrated filters. The basic fabrication process of the improved device resembled the conventional process used for the device without filters^22^. Opening areas of 16 mm^2^ were fabricated for integrating filters. Two opening holes were prepared (Fig. 2). Subsequently, filter sheets (Φ 0.4 µm polyethylene terephthalate sheet or Φ3.0 µm polycarbonate sheet) were cut into 4 mm squares by a carbon dioxide (CO_2_) laser. The filter sheets were transparent and suitable for microscopic observation. PDMS, with a prepolymer ratio of 15:1, was applied as a glue to the periphery of the opening hole. The filter sheets were aligned and bonded on the openings of the device structure by heating at 100 °C for 1 h on a hotplate.

### Experimental setup

**Figure 3:**
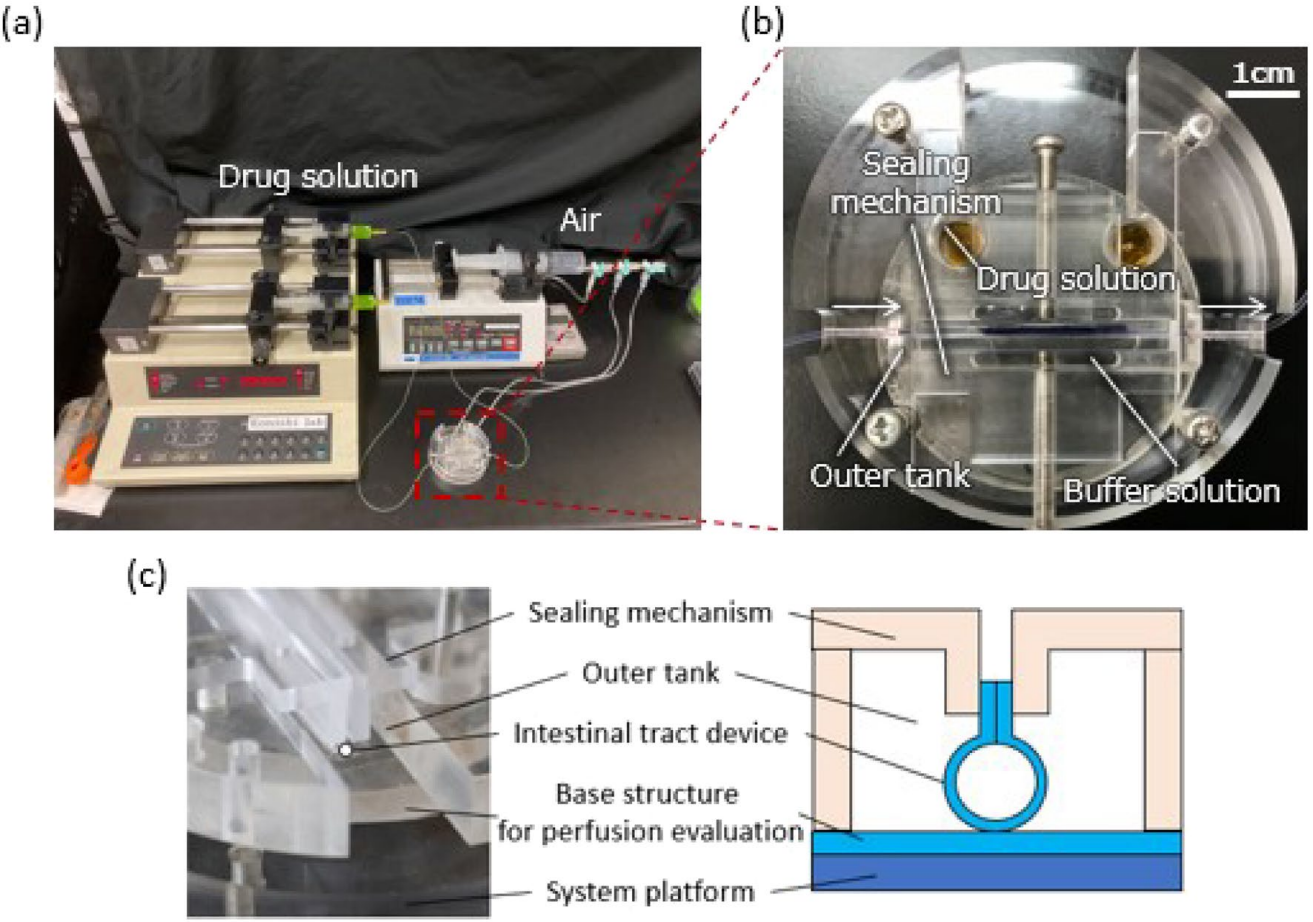
Experimental setup for the intestinal tract device system. (**a**) Entire experimental setup. Peripheral instruments for driving PBAs and introducing drug solution are shown. (**b**) Magnified top view of the device system. (**c**) Side view of the device system. The system is composed of a system platform, base structure for perfusion evaluation, intestinal tract device, and sealing mechanism. The sealing mechanism is used for maintaining the tubular state of the device. A buffer solution is filled in the outer tank for sampling the drug permeated through the filters.

Figure 3 shows the experimental setup of the intestinal tract device system for a substance permeation test. Figure 3a depicts a cross-section of the full setup, including peripheral instruments for driving PBAs and introducing the solution, and Fig. 3b depicts the magnified top view of the device system. The device system consisted of a base structure for perfusion evaluation, an outer tank, an intestinal tract device, and a sealing mechanism, which were all mounted on the acrylic system platform. The base structure was constructed using a 3D printer (AGILISTA, Keyence, JAPAN). A sealing mechanism was used at the top to maintain the tubular state of the device. A buffer solution was added to the lower reservoir and outer tank, for sampling the filtered solution.

The PBAs integrated into the device were pressurized and bent using a syringe pump (YSP-201, YMC) to form a tubular structure for the perfusion test. The PBAs were used to close and open the tubular structure, whereas a peripheral sealing mechanism was used to hold the tubular structure by clamping the top joint. The sealing mechanism, working in a similar manner to a vise, is shown in Fig. 3b. The mechanism has two parallel jaws, moved by screws, that clamp the top joint part of the device.

The solution for the perfusion test was introduced into the tubular structure using a syringe pump (PUMP33, Harvard Apparatus, Holliston, Massachusetts, USA), which was connected to the device by tubing. An outer tank filled with Hank’s Balanced Salt Solution (HBSS) buffer (Sigma #H1387 1 × 10 L, Sigma-Aldrich, USA) was prepared for penetrated liquid storage (Fig. 3b). HBSS (500 µL) was used in the perfusion test in this study. The fluorescence intensity of the sample was measured using a plate reader (Synergy HTX, BioTek, Winooski, Vermont, USA). Uranine (250 μM, Sigma-Aldrich) was used as a fluorescent reagent. The concentration-fluorescence intensity calibration curve was calculated using measured fluorescence intensity of Uranine whose concentration was adjusted in advance. The fluorescence intensity was converted into the Uranine concentration by using the obtained calibration curve.

### Cell culture and evaluation

Madin Darby canine kidney (MDCK-II) cell line (CRL-2936™) was purchased from American Type Culture Collection (Manassas, VA, USA) and cultured in Dulbecco’s modified Eagle’s medium (DMEM), supplemented with 10% heat-inactivated fetal bovine serum, 1% antibiotic–antimycotic, 1% nonessential amino acids, 2 mM l-glutamine, and 100 IU/mL penicillin/streptomycin. Cells were maintained at 37 °C in a 5% CO_2_/95% air mixture. MDCK-II cells were prepared for the solution permeation test. MDCK-II cell suspension (500 μL; approximately 1 × 10^5^ cells/mL) was introduced to the reservoir.

Cells were observed and evaluated using a phase contrast microscope (CKX41, Olympus). In parallel, the status of cells on the device was electrically evaluated, in addition to permeability by considering future TEER measurements^25–28^. Electrical impedance through cultured cells was estimated using a pair of electrodes: one was an upper electrode, introduced into the tubular structure, and the other was a lower electrode, placed outside the tubular structure. The electrode consisted of silver/silver chloride (Ag/AgCl) ink on a polymer substrate. A 0.5 μm thick Parylene C (poly[chloro-p-xylylene]) coating was added using a chemical vapor deposition (CVD) machine (Lab-coater PDS2010, Cookson Electronics Equipment, Providence, Rhode Island, USA). The coating was added to insulate and define an exposed detecting area. The upper electrode consisted of a silicon (Si) slip (4 mm × 58 mm × 100 μm), coated with Ag/AgCl (4 mm × 500 μm × 100 μm) at its tip, penetrating an acrylic chip. The acrylic chip was designed to be mounted and fixed on a structure for the sealing mechanism. The protruding electrode was designed to be inserted into a tubular device for detection. The lower electrode was integrated into a base structure, consisting of a lower reservoir with inlet and outlet channels. The lower reservoir enabled the supply of a fixed medium volume to the outside of the tubular intestinal tract device during measurement. The Ag/AgCl electrode was placed in the reservoir and wired for signal processing. The lower reservoir was filled with red buffer medium. The perfusion flow of 0.05 mL/min was introduced into the tubular device without causing any leak for a maximum of 60 min. Thus, electrical monitoring of the status of cells on the device was possible using the improved device.

## Results and discussion

This study presents the implementation of a permeable filter onto our previously developed openable artificial intestinal tract device. The permeable filters are integrated as additional functions, without affecting the performance of the bending PBAs, as shown in Fig. 1. Cells are cultured on a flat device (Fig. 1a), and cell growth is confirmed by microscopy. The tubular state achieved by the bending PBAs allows perfusion tests to be completed (Fig. 1b). Cells can be observed in a flat state by opening the device (Fig. 1c). The permeability of the newly integrated filter was evaluated using a perfusion test. In the present study, we cultured MDCK-II cells instead of Caco-2 cells on the device. The status of cells on the device and permeability will be electrically evaluated by considering future TEER measurement^25–28^.

### Fabrication of the intestinal tract device with integrated filters

**Figure 4:**
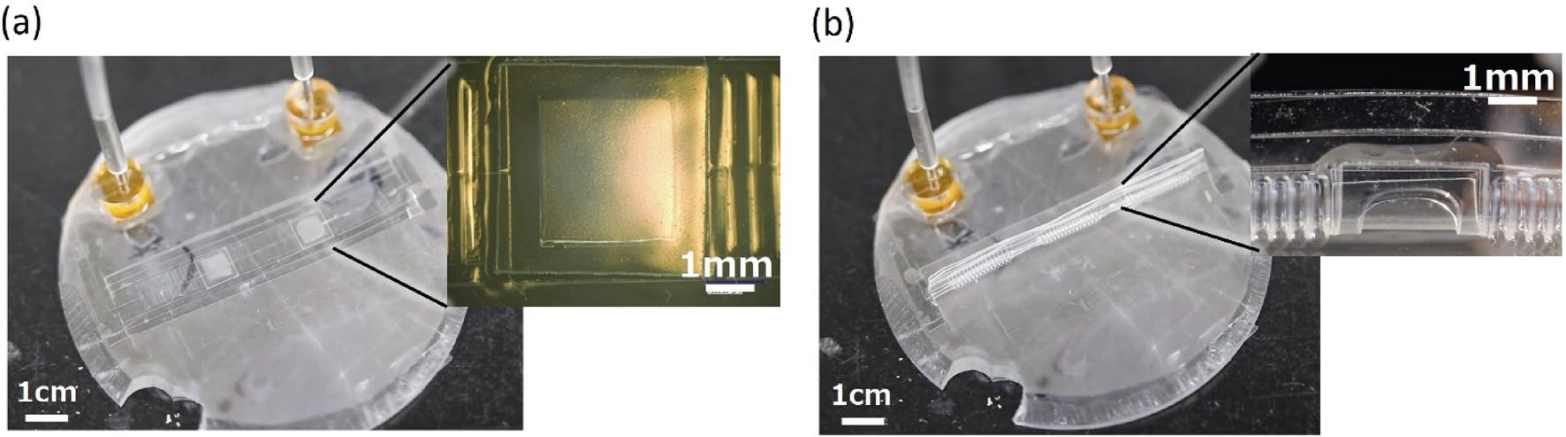
Fabrication and operation results of the artificial intestinal tract device with filters for drug permeation. (**a**) Fabricated device in the initial flat state. A magnified photograph of the integrated filter is also shown. (**b**) Tubular shape transformed by bending PBAs. The applied pressure for the device is 100 kPa; the tubular shape can be formed at no minimum 65 kPa. A magnified photograph of the integrated filter in the tubular structure is also shown.

Figure 4a shows the results of fabrication of the intestinal tract device with integrated filters on a system platform. The tubular device was approximately 1 mm in diameter and 13 mm long. Two square filters were integrated between the PBAs on the intestinal tract device. A magnified photograph of the filter is shown in Fig. 4a. PBA arrays were arranged on both sides of the filter. The device was 37.6 mm long, the valid area of the filter was 2.75 mm × 3 mm, and each PBA was 400 μm × 3.6 mm.

Shape transformation by PBAs between flat and tubular states was confirmed (Fig. 4b). The additive structures of filters showed no apparent influence on the shape transformation. The tubular shape could be formed at minimum 65 kPa. The applied pressure for the device in Fig. 4b was 100 kPa.

### Permeation evaluation through perfusion test

**Figure 5:**
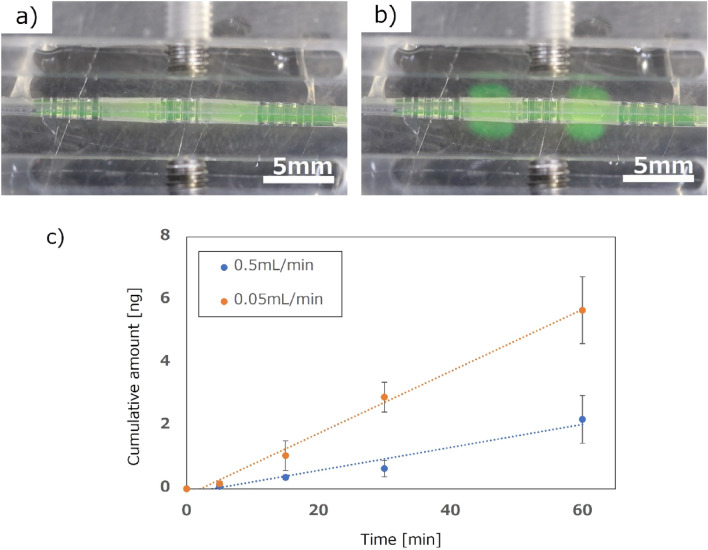
Permeation evaluation through perfusion test. (**a** and **b**) Time-lapse photographs of device during permeation and perfusion test. (**c**) Characteristics between flow velocity and permeability in cell-free perfusion experiment (n = 3). Each value represents the mean ± S.D. (n = 3). Two different flow velocity conditions of 0.05 and 0.5 mL/min were evaluated.

Permeation characteristics between flow velocity and permeability were evaluated in a cell-free perfusion test. Figure 5a,b show photographs of permeation and perfusion test. Uranine (2.5 μM, Sigma-Aldrich) was used as a fluorescent reagent to estimate the amount of transmission. HBSS buffer in the outer tank was sampled in volumes of 50 µL at 5-, 15-, 30-, and 60-min intervals. Calcein-AM exuded through the filter part as shown in Fig. 5b. The fluorescence intensity of the sample was measured and converted from the calibration curve to the cumulative transmission amount. Two different flow velocity conditions of 0.05 and 0.5 mL/min were evaluated using three devices for each condition. The average value from the three devices was calculated for permeation characterization. Figure 5c shows the results of permeation characterization. The amount of transmission showed an evident difference depending on the difference in flow velocity. The transmission amount through the filter to the outer tank decreased when the flow velocity increased in the tubular device. The results showed good alignment with the results reported by other studies^25–28^ and confirmed that the developed device equipped with filters was suitable for a solution permeation test.

### Cell culture on the device

**Figure 6:**
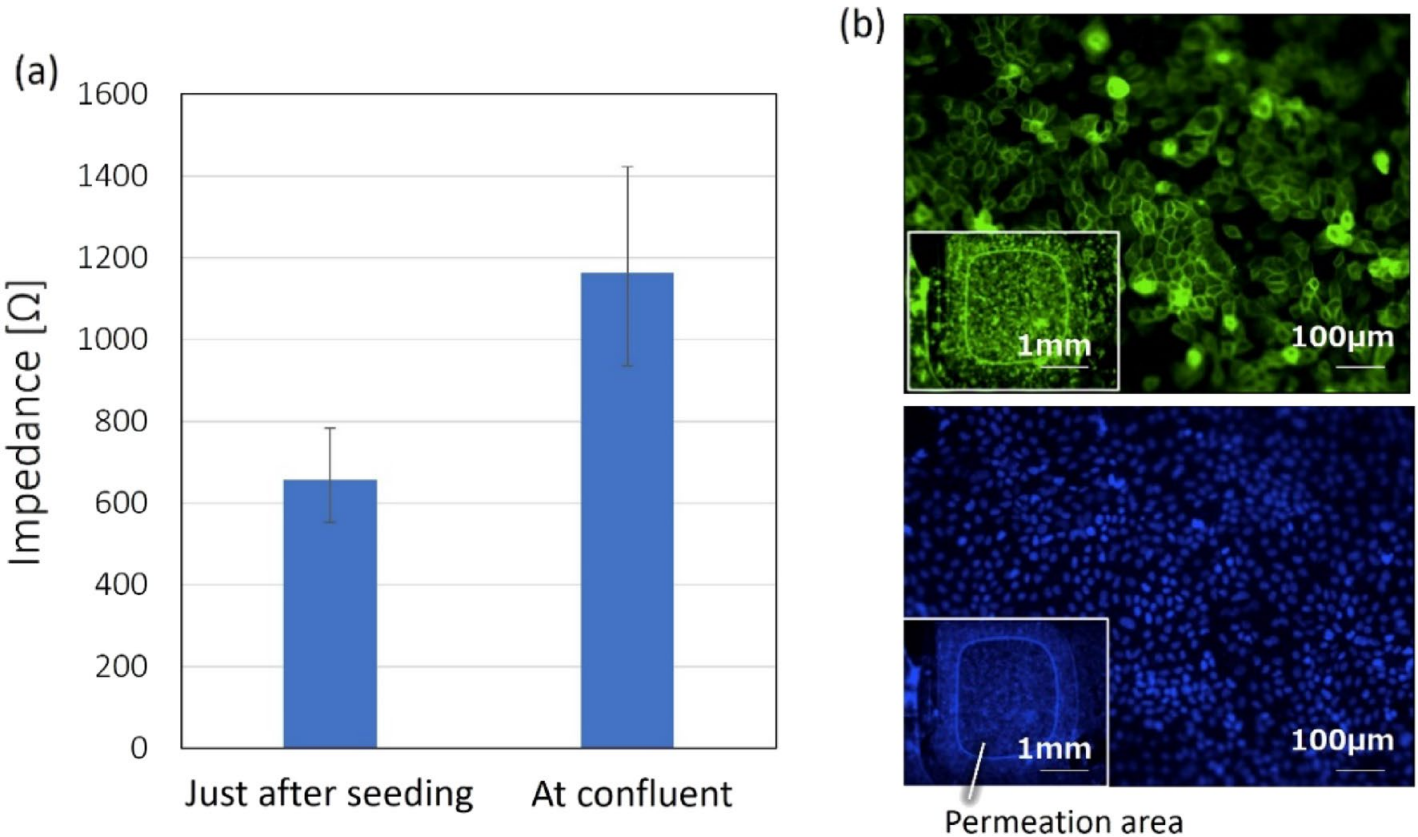
Impedance measurement results for MDCK-II cells on the device (n = 3), and phase difference at the time of measurement. Initial impedance at cell seeding and at a confluent state are evaluated and compared. An impedance increment of 500 Ω is converted to a TEER increase of approximately 74 Ω * cm^2^, since the filter area is 0.147 cm^2^. Fluorescence observation of MDCK-II cells in the confluent state was performed using a fluorescence microscope. The staining image using Actin shows the structure of the cytoskeleton and that using DAPI shows the nucleus of cells.

MDCK-II cells were cultured on the developed device for evaluating cell viability^29,30^. MDCK cells were cultured on a flat state of the device in a static condition for eleven days before reaching confluency. Images of cultured MDCK-II cells were captured using a phase contrast microscope, as shown in Fig. 6. In parallel, electrical impedance between electrodes was measured on a hot plate with a fixed temperature of 37 °C. An alternating current of 100 Hz frequency was applied using an LCR meter. The average value of the three samples was calculated. Figure 6 shows the results of impedance measurements of MDCK-II cells cultured on the device. Additionally, initial impedance at cell seeding and that at a confluent state were evaluated and compared. The initial impedance was approximately 600 Ω, whereas that at a confluent state reached almost 1200 Ω. The impedance was calibrated by the permeation area (0.147 cm^2^). Thus, we could confirm that the impedance increased depending on cell growth. Fluorescence observation of MDCK-II cells in the confluent state was performed using a fluorescence microscope (BZ-X710, Keyence). Since Actin is the main component of the cell matrix, it is possible to observe the structure of the cytoskeleton by staining. DAPI binds strongly to DNA, so it is possible to stain the nucleus of cells.

### Fluorescent reagent permeation on the device with cultured cells

**Figure 7:**
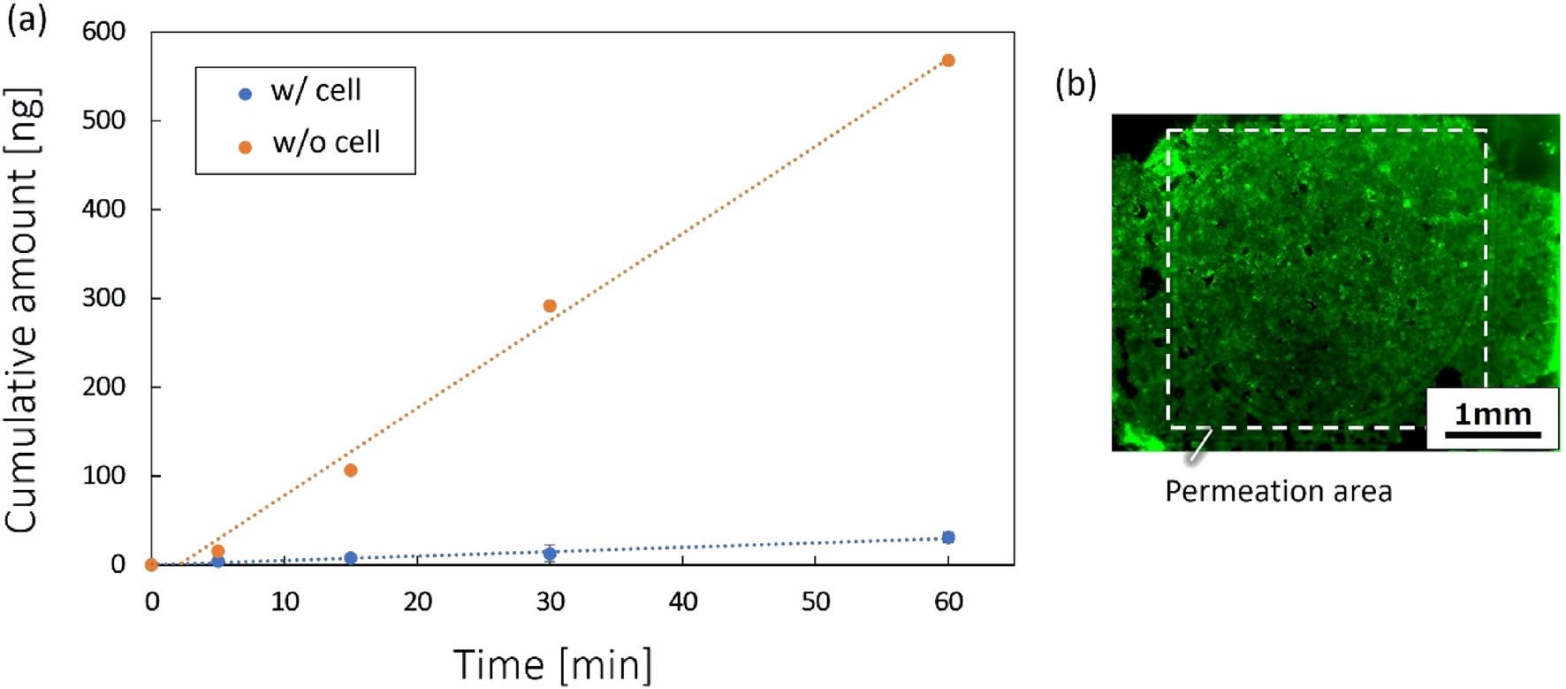
Evaluation results of permeation amount on the device. (**a**) Results of fluorescent reagent permeation experiment with and without MDCK-II cells (n = 3). Each value represents the mean ± S.D. (n = 3). (**b**) Cell-stained image after perfusion experiment. The flow velocity was set at 0.05 mL/min. Uranine (250 μM, Sigma-Aldrich) was used as a fluorescent reagent in the experiment. The reagent permeation on the device without cells in Fig. 5 is compared as control data, by taking into account the reagent concentration difference.

A fluorescent reagent permeation test was performed on the device with cultured cells, based on the cell-free perfusion test (Fig. 5). Figure 7a shows the permeation results with cultured MDCK-II cells. The reagent permeation on the device without cells was considered as control data in Fig. 7a. The amount of transmission decreased due to the existence of cells. Therefore, we increased the concentration (100 times) of reagent in evaluating the permeation with cells for Fig. 7. Uranine (250 μM, Sigma-Aldrich) was used as a fluorescent reagent in the experiment. We measured the amount of transmission through cells under the high concentration of reagent, whereas the data without cells in Fig. 7 were converted from those in Fig. 5 by accounting for the difference in reagent concentration. The amount of permeation with cells was reduced to approximately 5.5%, relative to that obtained without cells. Living cells on the device filter were confirmed immediately after the permeation test (Fig. 7b). Calcein-AM was used to stain living cells on the filter. The reduction in permeation was, therefore, caused by living cells on the filter. Consequently, the addition of filters enabled the openable intestinal tract device to evaluate fluorescent reagent permeation through cells.

## Conclusions

This paper presents the integration of permeable filters into an openable artificial intestinal tract device, which has been previously used for the estimation of drug absorption by cells cultured on its internal wall. The integration of filters enabled drug permeation and absorption analysis. Filter sheets were integrated into the device openings, which were approximately 1 mm in diameter and 13 mm in length. The developed device was used for characterization of permeation through the filters and for evaluation of fluorescent reagent permeation via cells cultured on the filter. It was confirmed that permeation through filters was dependent on the perfusion flow rate in the tubular device. The fluorescent reagent permeation was confirmed to be dependent on cell growth on the filters. These promising results indicate the possibility that drug permeation can be evaluated using the openable artificial intestinal tract device equipped with filters. Implementation of additional functions may still be improved because the current device uses assembled filters, which restrict production throughput.

## Data Availability

All data generated or analyzed during this study are included in this published article.
